# CXCR4 peptide-based fluorescence endoscopy in a mouse model of Barrett’s esophagus

**DOI:** 10.1186/s13550-021-00875-7

**Published:** 2022-01-10

**Authors:** Sabrina Marcazzan, Marcos J. Braz Carvalho, Matthias Konrad, Julia Strangmann, Anna Tenditnaya, Theresa Baumeister, Roland M. Schmid, Hans-Jürgen Wester, Vasilis Ntziachristos, Dimitris Gorpas, Timothy C. Wang, Margret Schottelius, Michael Quante

**Affiliations:** 1 II Medizinische Klinik, Klinikum rechts der isar, Technische Universität München, Munich, Germany; 2 Chair of Biological Imaging, School of Medicine, Technische Universität München, Munich, Germany; 3 Helmholtz Zentrum München, Institute of Biological and Medical Imaging, Neuherberg, Germany; 4 Institut für Pharmazeutische Radiochemie, Technische Universität München, Munich, Germany; 5 Division of Digestive and Liver Diseases, Columbia University Irving Medical Center, New York, NY USA; 6 Translational Radiopharmaceutical Sciences, Departments of Nuclear Medicine and Oncology, Centre Hospitalier Universitaire Vaudois, Lausanne, Switzerland; 7 Innere Medizin II, Universitätsklinik Freiburg, Universität Freiburg, Freiburg im Breisgau, Germany; 8 Present Address: Christian Doppler Laboratory for Viral Immunotherapy of Cancer, Medical University of Innsbruck, Peter-Mayr-Straße 4b, 6020 Innsbruck, Austria

**Keywords:** Esophageal cancer, Barrett’s esophagus, Dysplasia, CXCR4, Peptide, Molecular imaging, Endoscopy, Fluorescence imaging, Animal models

## Abstract

**Background:**

Near-infrared (NIR) fluorescence imaging has been emerging as a promising strategy to overcome the high number of early esophageal adenocarcinomas missed by white light endoscopy and random biopsy collection. We performed a preclinical assessment of fluorescence imaging and endoscopy using a novel CXCR4-targeted fluorescent peptide ligand in the L2-IL1B mouse model of Barrett’s esophagus.

**Methods:**

Six L2-IL1B mice with advanced stage of disease (12–16 months old) were injected with the CXCR4-targeted, Sulfo-Cy5-labeled peptide (MK007), and ex vivo wide-field imaging of the whole stomach was performed 4 h after injection. Before ex vivo imaging, fluorescence endoscopy was performed in three L2-IL1B mice (12–14 months old)  by a novel imaging system with two L2-IL1B mice used as negative controls.

**Results:**

Ex vivo imaging and endoscopy in L2-IL1B mice showed that the CXCR4-targeted MK007 accumulated mostly in the dysplastic lesions with a mean target-to-background ratio > 2. The detection of the Sulfo-Cy5 signal in dysplastic lesions and its co-localization with CXCR4 stained cells  by confocal microscopy further confirmed the imaging results.

**Conclusions:**

This preliminary preclinical study shows that CXCR4-targeted fluorescence endoscopy using MK007 can detect dysplastic lesions in a mouse model of Barrett’s esophagus. Further investigations are needed to assess its use in the clinical setting.

**Supplementary Information:**

The online version contains supplementary material available at 10.1186/s13550-021-00875-7.

## Introduction

Barrett’s esophagus (BE), which is highly associated with gastroesophageal reflux disease (GERD), is a precursor lesion of esophageal adenocarcinoma (EAC). Its prevalence is estimated at 0.5–2% in the general population and at 5–15% in symptomatic GERD-patients [1, 2]. However, only 0.1–0.5% of patients, diagnosed with BE, actually develop EAC [3, 4]. Despite its low incidence, EAC is associated with poor survival and the diagnosis at an early and curable stage is of primary importance [5–7]. Therefore, novel imaging systems are needed in order to improve the prognosis of EAC, also allowing a risk stratification for the progression from non-dysplastic BE to EAC.

In this context, the chemokine receptor 4 (CXCR4) has emerged as a promising marker for imaging. CXCR4 is overexpressed in different solid tumors, including EAC [8–11]. We previously showed that CXCR4 was mostly expressed at high levels on immune cells and epithelial cells and its expression correlated with progression from BE to dysplasia to EAC in the L2-IL1B mouse model of BE and in EAC patients [9]. Additionally, we used Cy5.5-conjugated anti-CXCR4 antibody (Ab) to visualize murine dysplastic lesions in situ [9]. However, high-affinity CXCR4-targeted fluorescent probes are needed to be able to accurately assess CXCR4 expression in such (pre)malignant lesions. In this preliminary study, we used a novel pentapeptide-based ligand design, based on the peptide scaffold successfully used in the [68 Ga] Pentixafor/Pentixather. Due to the superior brightness of the disulfonated analog of the cyanine dye Cy5 (“sulfo-Cy5”, *λe*_x,max_/*λ*_em,max_ in PBS: 649/666 nm) and the improved tissue penetration compared with conventional near-infrared dyes such as ICG [12], we chose to integrate this fluorescent label into the CXCR4-targeted peptide ligand design. For this purpose, we here conducted an evaluation of a fluorescent peptide-based probe for CXCR4 in a mouse model of BE by *ex-vivo* wide-field imaging and endoscopy. The goal would be to use such a fluorescent CXCR4-targeted peptide during live endoscopy to allow targeted biopsies for better dysplasia detection and thus patient surveillance.

## Methods

### Mice

All experiments on animals were performed following protocols approved by the Regierung Oberbayern in concordance with the German Animal Welfare Act and Ethical Guidelines of the Klinikum rechts der Isar, Technical University of Munich (TUM). L2-IL1B mice over-expressing IL1b under the control of EBV-L2 were generated as previously described [13, 14]. Mice were backcrossed to C57BL/6J mice, weaned at 21 days and fed with water and standard food (V1124-000, Ssniff, Soest, Germany) ad libitum*.*

### Histology

Mice were sacrificed by anesthetic overdose and the stomach and esophagus were fixed in 4% PFA, embedded in paraffin blocks and cut in sections for H&E staining. For microscopic scoring of lesions, H&E slides were used and the histopathological evaluation was performed by an expert gastroenterologist (MQ). The grade of inflammation, metaplasia and dysplasia was evaluated as previously reported [14].

### Peptide synthesis and characterization

The synthesis and characterization of the CXCR4-targeted peptide MK007 conjugated with Sulfo-Cy5-dye are described in the Additional file 1: Methods.

### Ex vivo imaging

Five 12-to-16-month-old IL1B mice were intravenously injected into the tail vein with 60 nmol of MK007 diluted in 0.9% NaCl (tot. vol. 150 µL; 400 nmol/mL) and sacrificed 4 h post injection (p.i.) by isoflurane overdosage. One non-injected 12-month-old IL1B mouse served as negative control. The whole stomach and esophagus were then excised and imaged using a homemade ex vivo wide-field fluorescence imaging system [15]. Briefly, the fluorescence images were acquired at different time exposure (0.2, 0.5, 1 and 2 s), using a 670 nm diode laser (B&W tek, Newark, DE, USA) and appropriate bandpass filter (780/10). A back illuminated EM-CCD camera (iXon DU888, Andor, Belfast, Northern Ireland, UK) was then used to capture the signal. The target-to-background ratio (TBR) of the stomach was performed by dividing the fluorescence signal in the squamocolumnar junction (SCJ) area (target lesion) with the adjacent normal gastric glandular tissue (background area) using ImageJ/Fiji software. In the organs, the signal quantification was performed by calculating the corrected total cell fluorescence/CTCF = integrated density – [(area * AVERAGE (mean background)]. For CTCF, three regions of interest (ROIs) without any tissue were used as background.

### Endoscopy

Three L2-IL1B mice (12–14 months old) were used for fluorescence molecular endoscopy before ex vivo imaging. Endoscopy was performed after sacrificing the mice at 4 h p.i. of MK007 using a homemade system previously developed by our group [16] and optimized for small animal imaging [17]. Briefly, the endoscopy system consisted of a flexible fiberscope (Micrendo-Fiberskop, SCHÖLLY FIBER OPTIC GMBH, Denzlingen, Germany) where both white light (provided by a 250-W halogen lamp, KL-2500 LCD, Schott AG, Mainz, Germany) and fluorescence (fiber coupled continuous wave (CW) laser diode emitting at 670 nm, SLD1332V, Thorlabs, Newton, NJ, USA) are coupled, allowing simultaneous color and near-infrared (NIR) fluorescence endoscopy. Videos in color and fluorescence channel were recorded using a custom-made endoscopy software and stored in AVI file format. Two NaCl-injected IL1B mice (17 and 12 months old) were used as negative controls for the validation of the endoscopy system. After endoscopy, the organs and whole excised stomach of all three MK007-injected-mice and one negative control (12 months old) were imaged as previously described. For quantification of Sulfo-Cy5 signal, single frames or frame sequences showing dysplastic lesions were used and the fluorescence signal from the target lesion and the background were quantified with ImageJ by handmade ROIs. Per each frame, the normal surrounding esophageal tissue without visible dysplastic lesions was considered as background. The TBR was then calculated by dividing the mean fluorescence intensity from the lesion by the one from the background region.

### CXCR4 immunofluorescence (IF) and confocal microscopy

After ex vivo imaging, the stomach and esophagus, spleen, liver, and kidneys were fixed in 4% PFA overnight at 4 °C and placed in 15% sucrose for 3–4 h at 4 °C. Tissues were then placed in 30% sucrose solution overnight at 4 °C and were then embedded in OCT on dry ice with isopropanol. Blocks were stored at − 80 °C and were transferred in − 20 °C overnight before cutting. Tissue sections (7 µm) were then cut with a cryotome (Thermoscientific, Waltham, MA, USA). For staining, slides were put at RT for drying overnight or at 37 °C for 1 h and stored at − 20 °C. Slides taken from − 20 °C were left few minutes at RT and staining for CXCR4 was performed as it follows: slides were washed twice in PBST (PBS 0.03% Triton X-100) and incubated with blocking and permeabilization solution (PBS 1.5% BSA 0.1% Triton X-100 10% goat serum) for 1 h RT. After that, rat anti-mouse CD184 Ab conjugated with PE (1:40, 2 h RT; #12–991-82, eBioscience, San Diego, CA, USA) was applied. Slides were then washed three times in PBST and mounted using Mounting Medium with DAPI (Vectashield, Vector Laboratories, Burlingame, CA, USA). After sealing the coverslips with nail polish, slides were stored at 4 °C protected from the light. Images were acquired by using Leica SP8 Lightning Confocal microscope (Leica Microsystems, Wetzlar, Germany). Positive and negative controls were included per each staining. Quantification of CXCR4 IF and Sulfo-Cy5 staining was performed using ImageJ/Fiji software by manual thresholding as per ImageJ instructions. Three to six fields at 40 × were used per each region (SCJ, squamous epithelium and normal gastric epithelium) and the positive area of each field was then measured in pixels and averaged per each region. Results are represented as single plotted mean value per each mouse and mean ± SEM of all mice.

### Statistical analysis

Quantitative data were statistically analyzed by unpaired T test with GraphPad Prism 9.0 software. *P* ≤ 0.05 was considered significant.

## Results

### Generation and in vitro characteristics of MK007

**Fig. 1:**
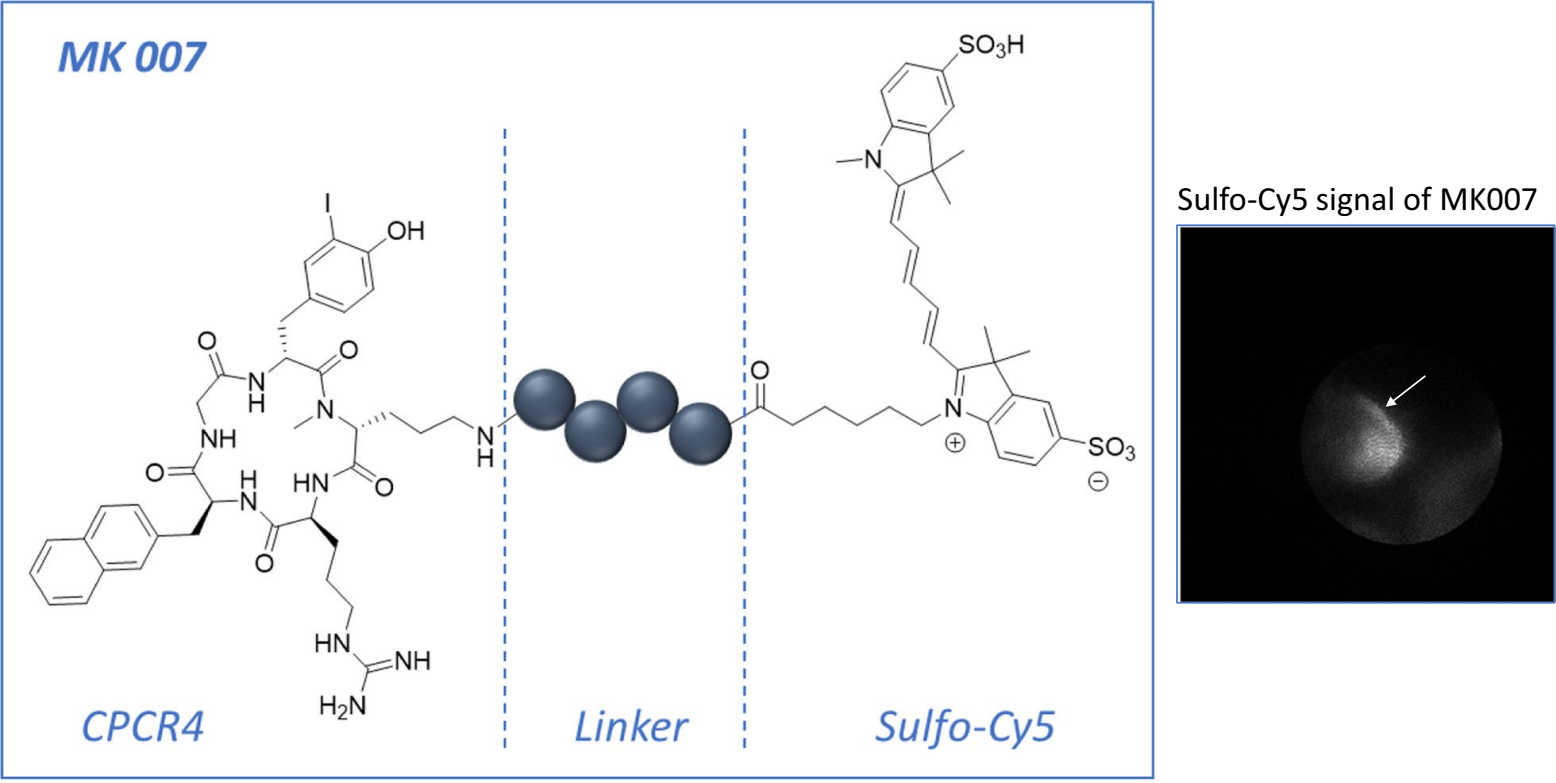
Structure (left) and Sulfo-Cy5 signal assessment (right) of the CXCR4-targeted fluorescent ligand MK007. For the assessment of fluorescence signal before mouse endoscopy, Sulfo-Cy5 MK007 1 mM was diluted in 0.9% NaCl to obtain a final concentration of 400 nmol/mL and measurement was performed using a 670 nm laser diode and a highly sensitive CCD camera. NaCl alone showed no fluorescence signal

Synthesis of the CXCR4-targeted cyclic pentapeptide CPCR4 was performed as previously reported [18, 19]. The peptide linker was prepared in analogy to a previously described manual SPPS procedure [20] and conjugated to generate the final Sulfo-Cy5-labeled peptide (MK007). Iodination of the de-protected and purified Cy5-bearing peptide was performed in analogy to a previously described procedure [21]. The peptide structure and assessment of Sulfo-Cy5 fluorescence signal before imaging are reported in Fig. 1.

### Ex vivo imaging and confocal microscopy of mouse stomach detect Sulfo-Cy5 in dysplastic lesions of IL1B mice

**Fig. 2:**
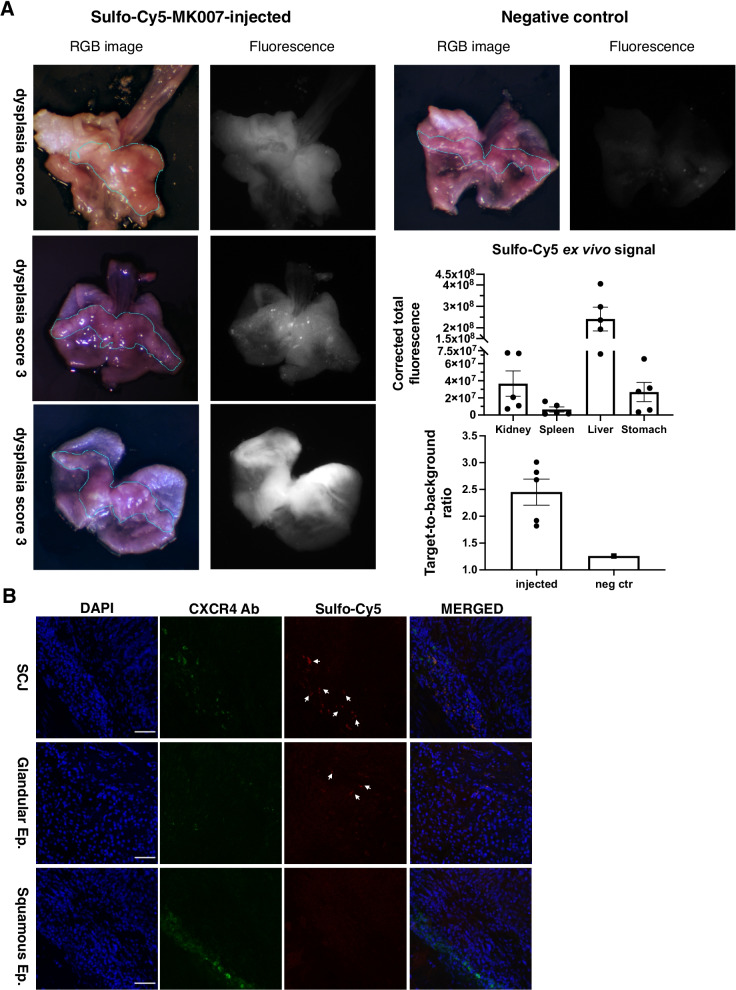
Ex vivo wide-field imaging shows accumulation of CXCR4-targeted MK007 in dysplastic lesions of IL1B mice. **A** Representative ex vivo fluorescence imaging of three IL1B mice injected with MK007 (left) and one non-injected 12-month-old IL1B mouse (right, upper row), which had a dysplasia score of 3. The different dysplasia score (1–3) is reported. The quantification of ex vivo* Sulfo-*Cy5 signal by CTCF in mouse stomach and organs 4 h post-injection showed peptide accumulation also in liver, kidney, and spleen (right, middle row). Target-to-background ratio (TBR) of injected mice was 2.45 ± 0.24 compared with negative control (1.26). TBR was calculated as reported in Methods. Data in both graphs are represented as mean ± SEM (*n* = 5). **B** Representative confocal images of areas from squamocolumnar junction (SCJ), squamous, and glandular epithelium of the IL1B mouse with dysplasia score 2 in 2A showed less or no Sulfo-Cy5 signal in non-dysplastic areas than dysplastic areas (arrows). Sulfo-Cy5 signal was detected by using Cy5.5 red channel. Additionally, most CXCR4 + cells were detected in the SCJ and squamous epithelium (Squamous Ep.) of IL1B mice, compared with the normal glandular epithelium (Glandular Ep.). Scale bars represent 50 μm

**Fig. 3:**
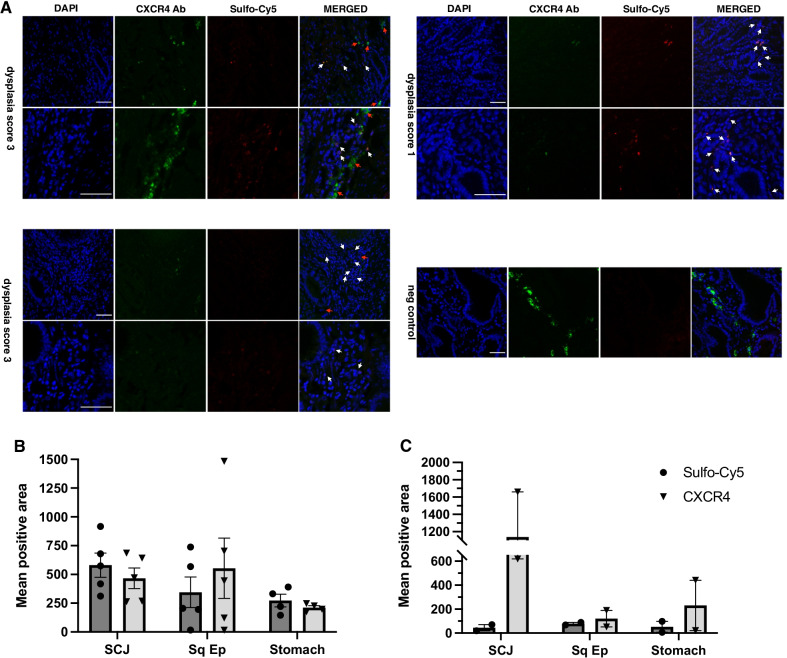
Co-localization of CXCR4 and Sulfo-Cy5 signal in lesions at the squamocolumnar junction of IL1B mice. **A** Representative confocal images of stomach of injected IL1B mice of different grade of dysplasia and of one negative control (12 months old). Orange/yellow fluorescence indicated the co-localization of MK007 signal with CXCR4 Ab (white arrows) next to dysplastic areas at the SCJ. In some CXCR4 + cells, no MK007 uptake was observed (red arrows). The staining for CXCR4 was performed with anti-CXCR4 Ab conjugated with PE as described in the Methods section. Sulfo-Cy5 signal was detected by using Cy5.5 red channel. Scale bars represent 50 μm. **B** Quantification of CXCR4 expression and Sulfo-Cy5 signal in SCJ, squamous epithelium (Sq Ep) and glandular epithelium (Stomach): in the SCJ, the mean CXCR4 and Sulfo-Cy5 signal were 580.6 ± 105.2 and 465.8 ± 89.19, respectively. A much lower CXCR4 expression (211.9 ± 15.74) and Sulfo-Cy5 signal (272.8 ± 54.99) were detected in the glandular epithelium. One mouse presented a high CXCR4 expression in the inflamed squamous epithelium but without an increase of Sulfo-Cy5 signal. Data are represented as single plotted values and mean ± SEM (*n* = 5). **C** No Sulfo-Cy5 signal (mean positive area < 100 pixels) was detected in negative controls (*n* = 2). Signal quantification was expressed by calculating the mean positive area in pixels per each mouse and data are represented as single plotted values and mean ± SEM

In order to evaluate the CXCR4-mediated accumulation of MK007 within dysplastic lesions in IL1B mice, ex vivo wide-field imaging was performed on the whole excised stomach and esophagus bearing dysplastic but also metaplastic (control) lesions at the same time. The age of the experimental animals was chosen based on the appearance of first symptoms indicating a late stage of disease such as weight loss and hunched position and the presence of macroscopically visible dysplastic lesions with high dysplasia grade in mice aged 12 months and over [13, 14]. The starting dose (60 nmol) and the time for evaluation of MK007 (4 h p.i.) was based on the stability and slow degradation rate of MK007 (data not shown), the measurement of fluorescence of Sulfo-Cy5-MK007 (Fig. 1 and Additional file 1: Fig. S1) and a preliminary evaluation of Sulfo-Cy5 signal by confocal microscopy 6 h p.i. (Additional file 1: Fig. S1). The ex vivo imaging after the injection of 60 nmol MK007 is showed in Fig. 2A: Sulfo-Cy5 signal was increased at dysplastic areas at the SCJ (blue line) compared with the normal glandular area or BE areas of the SCJ (control). This relative increase in MK007 accumulation into the stomach of IL1B mice was further confirmed by confocal microscopy (Fig. 2B, arrows). Indeed, IF staining for CXCR4 confirmed that MK007 mostly localized in regions with high densities of CXCR4 + cells (Fig. 3A). Quantification of CXCR4 IF and Sulfo-Cy5 signal reported in Fig. 3B and C clearly shows that the highest signals from CXCR4 and/or Sulfo-Cy5 were located at the SCJ, while the stomach displayed a low signal. The peptide biodistribution in liver, spleen and kidneys of IL1B mice (Additional file 1: Fig. S2) was similar to those observed in other mouse models of cancer after intravenous injection (data not shown). In conclusion, ex vivo imaging and confocal microscopy in IL1B mice showed that MK007 primarily accumulated in cells present next to dysplastic lesions in the stomach, while a minimum Sulfo-Cy5 signal was observed in the normal stomach tissue.

### MK007 is detectable by fluorescence endoscopy in dysplastic regions at the SCJ of IL1B mice

**Fig. 4:**
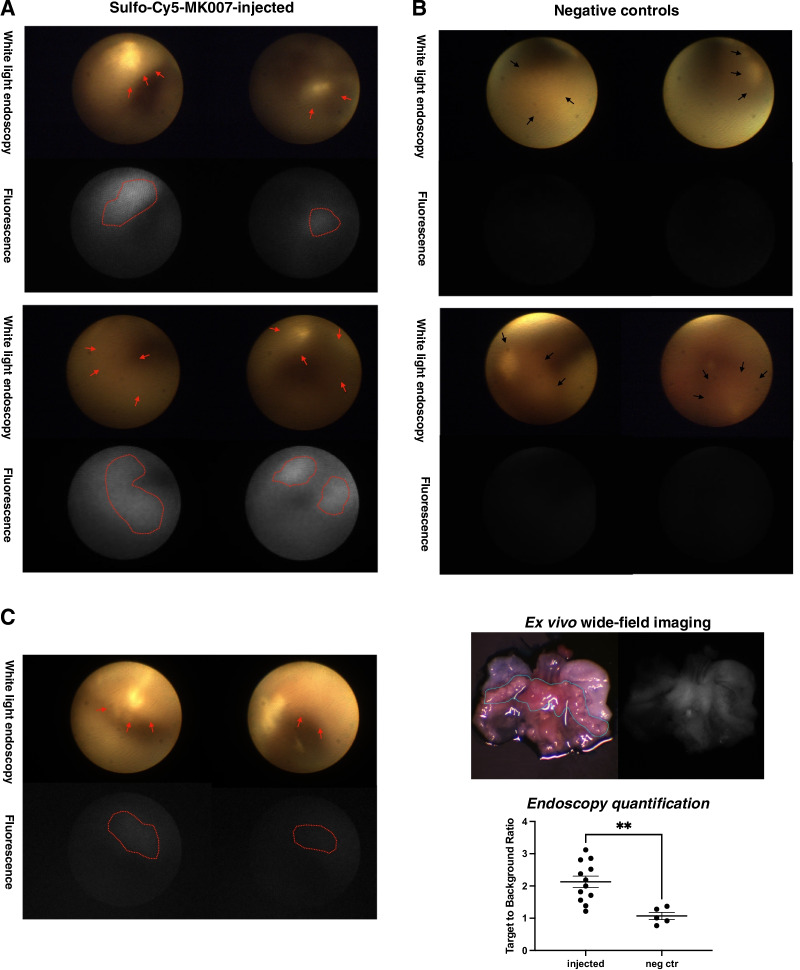
Dysplastic lesions of IL1B mice detected by fluorescence endoscopy and MK007. **A** Representative endoscopy images of two MK007-injected IL1B mice (60 nmol; 4 h p.i.), 14 and 12 months old (left) and two negative controls, 17 and 12 months old (right). Two endoscopy frames per each mouse are showed: visible dysplastic lesions in the color channel (red arrows) corresponded with the presence of Sulfo-Cy5 signal (red line). No Sulfo-Cy5 signal was detected in negative controls, despite visible lesions were found in both mice (black arrows). Fluorescence images are showed in grey scale*.* **C** Endoscopy images (left) and ex vivo wide-field imaging (right, up) of the third injected mouse, 14 months old. The ex vivo imaging of the other two injected mice is showed in Fig. 2A. Sulfo-Cy5 signal observed by endoscopy is indicated by the red line. A high Sulfo-Cy5 signal in the SCJ (blue line) was further confirmed by ex vivo imaging of the whole stomach. Quantification of Sulfo-Cy5 signal from all lesions in injected mice showed a significantly higher TBR than lesions in negative controls. Two to five dysplastic lesions were evaluated per each mouse. The signal quantification was performed as described in the Methods section. Data are represented as the TBR of each evaluated lesion and mean value ± SEM. **P < 0.01 by unpaired two-tailed T test

**Fig. 5:**
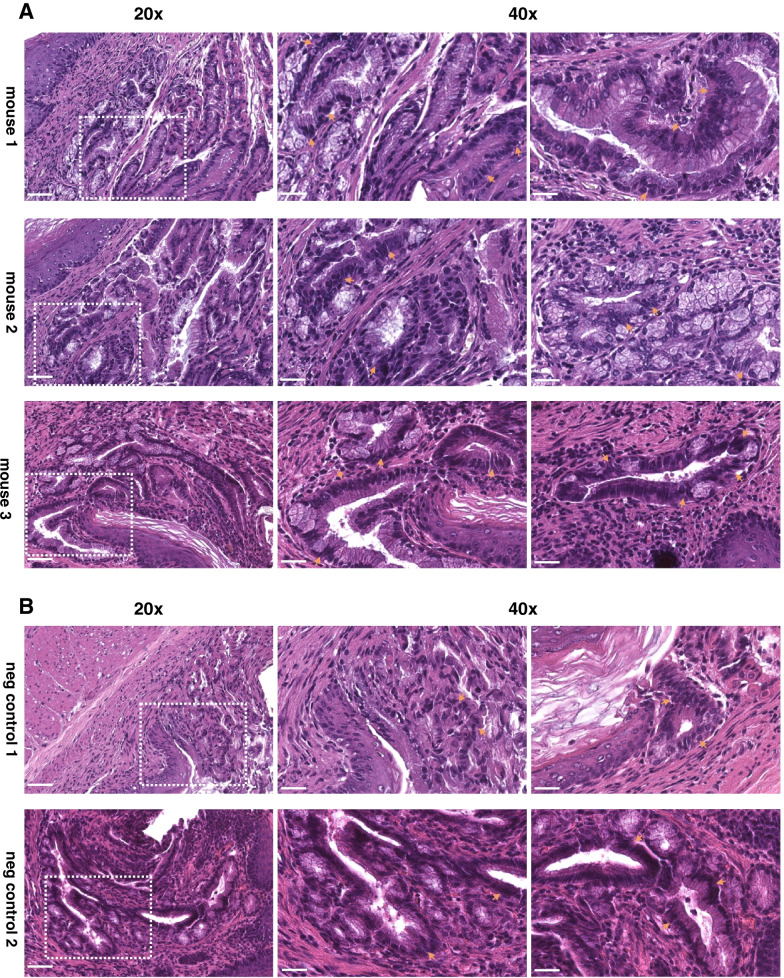
Histopathological confirmation of dysplastic lesions in IL1B mice. **A** Representative H&E images of all three MK007-injected IL1B mice and evaluated by endoscopy: the dysplastic lesions were located at the junction between the squamous epithelium and columnar epithelium. At a higher magnification, the lesions displayed a dysplasia score of 3 out of 4 (orange arrows) in all the three mice. Metaplasia and infiltration of inflammatory cells such as lymphocytes were also present. **B** Representative H&E images of the two negative control IL1B mice used for endoscopy: both negative control mice presented metaplastic or dysplastic lesions (arrows) at the junction between the squamous and the columnar epithelium but only one mouse (negative control 2) had dysplasia (score 3). The other mouse (negative control 1) had no dysplasia (score 0). Scale bars represent 50 μm (20 x) and 25 μm (40 x)

Before doing ex vivo wide-field imaging, fluorescence molecular endoscopy was performed on three IL1B mice (one 12 months old and two 14 months old; Fig. 4A, C, left) injected with MK007 to evaluate the potential of imaging CXCR4 in BE. Two NaCl-injected IL1B mice (12 and 17 months old with visible dysplastic lesions) were used as negative controls for endoscopy (Fig. 4B). Ex vivo imaging was performed on all three injected mice and only one negative control 12-month-old-mouse (Figs. 2A, 4C, right). Figure 4 shows that MK007 preferentially accumulated in the dysplastic lesions of all the three injected mice evaluated by endoscopy (red arrows and lines), while no Sulfo-Cy5 signal was detected in negative controls. Quantification of the Sulfo-Cy5 signal (Fig. 4C, right) shows a statistically significant higher mean TBR in injected mice (*P* = 0.0023) than negative controls, corroborating the validity and the specificity of our novel molecular endoscopy system. The dysplastic lesions of all three mice evaluated by endoscopy were further confirmed by histological analysis (Fig. 5). Altogether, our preliminary results showed the feasibility of detecting dysplastic lesions in IL1B mice by using the CXCR4-targeted peptide-based probe MK007 and the validity of our fluorescence endoscopy system.

### IC50 of radiolabeled counterpart of MK007 for human CXCR4

Given promising data obtained with MK007 in our preclinical mouse model, we performed preliminary in vitro studies on human cell lines. Competition binding studies (IC50) were performed [22] using Jurkat cells (4 × 10^5^ cells/sample) and [^125^I]FC131 as radioligand. The ^125^I-labeled, chemically identical counterpart of MK007 showed a log *P* of − 1.83 ± 0.02 at pH 7.4 (PBS). In a competitive binding assay using Jurkat human T-cell leukemia cells and [^125^I]FC-131 as the radioligand, MK007 demonstrated high CXCR4 affinity with an IC_50_ of 10.2 ± 4.0 nM (*n* = 3).

## Discussion and conclusions

The increasing incidence of EAC and the poor survival of patients affected demand the development of novel strategies for early detection. Indeed, white light endoscopy and random biopsy collection have resulted in the miss diagnosis of more than 50% of EAC cases [23]. Among other promising endoscopy systems, NIR fluorescence-based endoscopy has been recently emerged as a potential strategy for effective early detection [24–26]. We report here for the first time the use of CXCR4-targeted endoscopy for dysplastic lesions in a mouse model of BE using the novel CXCR4-targeted Sulfo-Cy5-labeled peptide-based probe MK007. The potential use of far-red endoscopy with fluorescent probes for dysplasia detection is supported by the statistically significant difference in the TBR between Sulfo-Cy5 injected mice (mean 2.13 ± 0.17) and NaCl-injected mice (mean 1.07 ± 0.11). To date, the CXCR4-targeted endoscopy imaging was reported only in a mouse model of bladder cancer, in which the fluorescent CXCR4 antagonist TY4003 was visualized in Carcinoma in Situ (CIS) lesions in chemically-treated mice but not in untreated mice [27]. In contrast to our study, the TBR of carboxyfluorescein-labelled TY4003 was not provided and the fluorescent peptide was applied topically instead of systemic [27]. Topical application may be of advantage because only a small amount of peptide will reach non-target or CXCR4-expressing organs (Additional file 1: Fig. S2). Despite CXCR4 staining co-localized with Sulfo-Cy5 (Fig. 3) at the SCJ of IL1B mice, future studies on Sulfo-Cy5-MK007 should consider the topical application of the probe. Indeed, the application of far-red and NIR fluorescent probes may be preferred for in vivo clinical imaging to green fluorescent probes as TY4003 due to lower tissue autofluorescence, higher penetration depth and specificity [24, 28, 29].

Our data on ex vivo imaging of the whole stomach and confocal microscopy after injection of Sulfo-Cy5 CXCR4-targeted peptide are consistent with our previous imaging of dysplastic lesions in IL1B mice [9], in which CXCR4 could detect dysplasia but not BE metaplasia. In that study, we injected an anti-CXCR4 Ab conjugated with Cy5.5 and evaluated the uptake in the SCJ by ex vivo imaging. CXCR4 expression was confirmed by IHC on fixed stomach and esophagus. [9]. In this study, we instead used IF for CXCR4 and confocal microscopy to directly detect the Sulfo-Cy5 signal from MK007 in SCJ and we observed in few cases CXCR4 + cells with no Sulfo-Cy5 signal. However, since the staining for CXCR4 in the stomach required permeabilization and cytoplasmic expression of CXCR4 has been described in other types of cancer [30], those CXCR4 + cells without peptide uptake may express CXCR4 only in the cytoplasm, while MK007 binds only to cells expressing CXCR4 on the membrane. Nevertheless, our previous data on CXCR4 expression in IL1B mice [9] showed that CD45 + CD11b + myeloid cells, neutrophils, and CD3 + T cells contributed to a high stromal CXCR4 expression in late-stage IL1B mice. Further characterization will be necessary to identify the CXCR4-high cells which did not internalize Sulfo-Cy5 MK007.

Although we did not inject MK007 and a CXCR4-blocker in IL1B mice [9], we found the same distribution of Sulfo-Cy5 MK007 signal by ex vivo wide-field imaging of the whole stomach (Fig. 2). Indeed, the use of fluorescent antibodies in patients has several significant drawbacks, including slow background clearance, delayed accumulation at the target site, and immunogenicity. Therefore, peptide-based fluorescent probes may be a valuable alternative due to their small size, low immunogenicity and cost, when used locally as intended for endoscopic procedures [31]. For this reason, our proof-of-concept study on the CXCR4-targeted Sulfo-Cy5-conjugated probe MK007 in IL1B mice encourage the further development of fluorescent ligands for endoscopic detection and surveillance, not only for patients with BE but also gastric cancer or colon cancer risk. Another preclinical study reported good results in using the CXCR4-specific peptide R-NIR750 conjugated with NIR fluorescent dye VivoTag-S750 for in vivo imaging of primary tumors and lung metastases [26]. However, endoscopy is necessary for esophageal cancer imaging, as the subcutaneous model does not reflect the dysplasia progression observed in patients [32].

Our proof-of-concept study also presents limitations: we performed endoscopy and imaging in a reduced number of mice and we did not perform further evaluation on human tissues, but rather focused on the murine CXCR4 receptor (target of this preliminary study) in a preclinical mouse of BE [33]. Indeed, the murine CXCR4 has limited homology with human CXCR4 and a modification of MK007 for human CXCR4 may be needed. Nevertheless, our data from in vitro studies and first clinical data on a patient with multiple myeloma (MM) using a new CXCR4-targeted Tc ligand (^99m^Tc; based on the structure of MK007) for SPECT/CT imaging [34] are encouraging for the potential clinical use of a modified MK007. Indeed, the ligand accumulated in intramedullary MM lesions and no high accumulation was observed in the liver, in contrast to mice [34]. Furthermore, we have not yet determined the affinity of MK007 for the murine CXCR4 receptor and the specificity of MK007 in the IL1B model. In contrast to our previous study, we did not inject the mice with a CXCR4 inhibitor plus MK007, but rather used NaCl-injected mice to further validate the specificity of the MK007 signal detected by our endoscopy system [17]. Additionally, we used only IL1B mice in a late stage of disease with an age variation based on the development of symptoms and large visible dysplastic lesions, needed for the present proof-of-concept study on MK007. We also evaluated only one time-point (4 h p.i.) which resulted in a fluorescence signal detectable by both endoscopy and ex vivo imaging. Future studies on MK007 should include IL1B mice with no dysplasia and low-grade dysplasia and different time points after injections. However, our preliminary results, showing significant MK007 uptake in regions with a high density of CXCR4-expressing cells, and previous data, which demonstrated high mCXCR4 affinity for similar compounds based on the same peptide-linker-label sequence and design as applied in MK007 [20] are highly indicative of mCXCR-mediated uptake of MK007. Additionally, we were able to specifically detect the Sulfo-Cy5 signal from MK007 in situ by endoscopy, as mice without Sulfo-Cy5-MK007 presented a significantly lower TBR.

In conclusion, our preliminary data show that dysplastic lesions can be detected by imaging using a CXCR4-targeted far-red-fluorescent probe. Further studies on excised human tissues and IL1B mice < 12 months are needed to evaluate the potential of CXCR4-targeted endoscopy and imaging for the identification of pre-dysplastic lesions in patients with BE.

## Supplementary Information

**Additional file 1.** Methods, Peptide synthesis, Determination of lipophilicity (log P), Determination of CXCR4 affinity.** Figure S1**. Evaluation of MK007 6 hours post-injection and MK007 fluorescence signal.** Figure S2**. Ex vivo imaging and confocal microscopy of main mouse organs after injection of MK007.

## Data Availability

The data that support the finding of this study are not publicly available due to patent ownership interests but are available from the corresponding author on reasonable request.

## Abbreviations

BE: Barrett´s Esophagus
GERD: Gastroesophageal reflux disease
EAC: Esophageal adenocarcinoma
CXCR4: Chemokine receptor 4
SCJ: Squamocolumnar junction
ROI: Region of interest
TBR: Target-to-background ratio
