# Supplementary material for: CXCR4 peptide-based fluorescence endoscopy in a mouse model of Barrett’s esophagus
Source: EJNMMI Res. 2022 Jan 10;12:2. doi: 10.1186/s13550-021-00875-7 (PMC8748556; doi:10.1186/s13550-021-00875-7)
Supplement: Supplementary file 1 — Additional file 1. Methods, Peptide synthesis, Determination of lipophilicity (log P), Determination of CXCR4 affinity. Figure S1. Evaluation of MK007 6 hours post-injection and MK007 fluorescence signal. Figure S2. Ex vivo imaging and confocal microscopy of main mouse organs after injection of MK007. [file 13550_2021_875_MOESM1_ESM.docx]

**Additional file 1**

**Supplementary Methods**

**Peptide synthesis**

The Fmoc-(9-fluorenylmethoxycarbonyl-) protected amino acids were purchased from either Bachem (Bubendorf, Switzerland) or Iris Biotech GmbH (Marktredwitz, Germany). 2-Chlorotrityl chloride (2CTC) resin and coupling reagents were also obtained from Iris Biotech GmbH (Marktredwitz, Germany) or from Sigma-Aldrich (Munich, Germany) or Molekula GmbH (Garching, Germany). Solvents and reagents for organic synthesis were purchased from either Alfa Aesar (Karlsruhe, Germany), Sigma-Aldrich (Munich, Germany) or VWR (Darmstadt, Germany). The Sulfo-Cy5 carboxylic acid was obtained from Lumiprobe (Hunt Valley, USA).

Analytical and semi-preparative HPLC was performed using a Shimadzu gradient system from Shimadzu Deutschland GmbH (Neufahrn, Germany), each equipped with a SPD20A UV/Vis detector (λ = 220 nm, 254 nm). A Multokrom 100 C18 (125 × 4.6 mm, 5 μm particle size; CS GmbH (Langerwehe, Germany)) column was used for analytical HPLC at a flow rate of 1 mL/min. Semi-preparative HPLC purification was performed using a Multokrom 100 RP 18 (250 × 10 mm, 5 μm particle size) column (CS GmbH (Langerwehe, Germany)) at a constant flow rate of 5 mL/min. For all HPLC analyses, gradients of acetonitrile (0.1% TFA, solvent B) in water (0.1% TFA, solvent A) were generally used as mobile phase.

*Synthesis of the CXCR4-targeted cyclic pentapeptide CPCR4*

The cyclic pentapeptide CPCR4 (*cyclo*(d-Tyr^1^-d-[NMe]Orn^2^-Arg^3^-Nal^4^-Gly^5^) was prepared according to a previously reported manual solid-phase peptide synthesis (SPPS) protocol [1, 2]. Upon removal of all acid labile protecting groups using TFA and precipitation in cold Et_2_O, the crude peptide was purified using semi-preparative HPLC: (10 – 95% B in 15 min): *t_R_* = 6.49 min, ĸ´= 4.01. Calculated monoisotopic mass (C_36_H_47_N_9_O_6_): 701.36, found: 701.8 [M+H$]^{+}$, 351.4 [M+2H$]^{2+}$.

*Synthesis of the linker unit*

The peptide linker was prepared in analogy to a previously described manual SPPS procedure [3]. The final product was cleaved from the resin support using a mixture of DCM/HFIP (4/1, v/v) for 2x30 min. Upon evaporation of the solvents, the crude peptide linker was purified using semi-preparative HPLC.

*Conjugation of the linker unit to CPCR4*

Conjugation of the peptide linker to CPCR4 was carried out in analogy to a previously described procedure [3]. Upon removal of the *N*-terminal Fmoc protecting group on the linker unit using piperidine in DMF (20/80, v/v), the solvents were evaporated under reduced pressure and the crude peptide-linker-conjugate was purified using semi-preparative HPLC.

*Functionalization with Sulfo-Cy5*

The Sulfo-Cy5-dye (free carboxylic acid, 1.5 eq.) was added to a solution of HOAt (1.5 eq.), TBTU (1.5 eq.) and DIPEA (3.0 eq.) in DMF and allowed to pre-activate for 15 min before the *N*-terminally deprotected CPCR4-linker construct was added. After completion of the coupling reaction (monitored by analytical HPLC), the reaction mixture was maximally concentrated *in vacuo*, and TFA was added. After 1.5h, the TFA was evaporated in N_2_-stream and the residual crude product was purified using semi-preparative HPLC. HPLC: (5 – 55% B in 15 min): *t_R_* = 8.73 min, ĸ´= 2.76. Calculated monoisotopic mass (C_87_H_111_N_19_O_17_S_2_): 1757.78, found: 880.5 [M+2H$]^{2+}$, 587.3 [M+3H$]^{3+}$.

*Solution-phase iodination of d-Tyr^1^, leading to the final product (MK007)*

Iodination of the de-protected and purified Cy5-bearing peptide was performed in analogy to a previously described procedure [4]. The desired mono-iodinated product was directly isolated from the reaction mixture via semi-preparative HPLC. HPLC (5 – 55% B in 15 min): *t_R_* = 11.00 min, ĸ´= 3.93. Calculated monoisotopic mass (C_87_H_110_IN_19_O_17_S_2_): 1883.68, found: 942.7 [M+2H$]^{2+}$, 630.4 [M+3H$]^{3+}$.

**Determination of lipophilicity (log P)**

To determine the log P of MK007, the corresponding non-iodinated precursor was radioiodinated using the Iodogen method, and the octanol/PBS partition coefficient of [^125^I]MK007 was determined using a modified shake-flask-method [5].

**Determination of CXCR4 affinity**

Competition binding studies (IC50) were performed as described [6], using Jurkat cells (4×10^5^ cells/sample) and [^125^I]FC-131 as radioligand. Experiments were performed in triplicate with n=3 per concentration in each experiment. IC_50_ values were calculated using GraphPad Prism 6.01 (Graph Pad Software, San Diego, USA).

**List of abbreviations**

DMF: Dimethylformamide; TFA: Trifluoroacetic acid; HPLC: High-performance liquid chromatography; DIPEA: *N*,*N*-Diisopropylethylamine; HOAt: 1-Hydroxy-7-azabenzotriazole; TBTU: 2-(1H-Benzotriazole-1-yl)-1,1,3,3-tetramethylaminium tetrafluoroborate; DCM: Dichloromethane; HFIP: Hexafluoro-2-propanol.

**Supplementary References**

1. Demmer, O., et al., *Introduction of functional groups into peptides via N-alkylation.* Org Lett, 2008. **10**(10): p. 2015-8.

2. Demmer, O., et al., *PET imaging of CXCR4 receptors in cancer by a new optimized ligand.* ChemMedChem, 2011. **6**(10): p. 1789-91.

3. Osl, T., et al., *A new class of PentixaFor- and PentixaTher-based theranostic agents with enhanced CXCR4-targeting efficiency.* Theranostics, 2020. **10**(18): p. 8264-8280.

4. Schottelius, M., et al., *An optimized strategy for the mild and efficient solution phase iodination of tyrosine residues in bioactive peptides.* Tetrahedron Letters, 2015. **56**(47): p. 6602-6605.

5. Schottelius, M., et al., *Improvement of Pharmacokinetics of Radioiodinated Tyr3-Octreotide by Conjugation with Carbohydrates.* Bioconjugate Chemistry, 2002. **13**(5): p. 1021-1030.

6. Gourni, E., et al., *PET of CXCR4 expression by a (68)Ga-labeled highly specific targeted contrast agent.* J Nucl Med, 2011. **52**(11): p. 1803-10.

**Supplementary Figures**

**Fig. S1**

Evaluation of MK007 6 hours post-injection and MK007 fluorescence signal**.** A) Representative confocal images of the stomach of a IL1B mouse injected with 15 nmol of MK007 and sacrificed 6 hours post-injection: despite more Sulfo-Cy5 signal was detected in the squamocolumnar junction (SCJ) than in squamous epithelium (sq. epithelium) and stomach, only a few cells at the SCJ presented Sulfo-Cy5 signal. B) Representative confocal images of the spleen and liver: a low Sulfo-Cy5 signal was observed in both organs. C) Measurement of fluorescence signal of high-dose MK007 by using a 670 nm laser diode and a highly sensitive CCD camera: NaCl showed no signal.

.

**Fig. S2**

Ex vivo imaging and confocal microscopy of main mouse organs after injection of MK007. A) Representative ex vivo fluorescence imaging of IL1B mice organs 4 hours after the injection of 60 nmol of MK007 diluted in 0.9% NaCl and the negative control. High Sulfo-Cy5 signal was observed in liver and kidneys of injected mice. The imaging of the stomach from these mice is reported in Figure 2A. Confocal microscopy of main organs from one representative injected mouse (same as Figure 2B) confirmed the *ex vivo* imaging results. Compared with the SCJ of IL1B mice, co-localization of Sulfo-Cy5 with CXCR4 was observed in few cells (arrows), suggesting a higher non-specific uptake of MK007 by those organs 4 hours post-injection. In contrast, the spleen presented a higher expression of CXCR4 and co-localization with MK007 signal was observed in more cells. Scale bars represent 50 μm.
